# Growing Interest in Microbiome Research Unraveling Disease Suppressive Soils against Plant Pathogens

**DOI:** 10.1264/jsme2.ME3304rh

**Published:** 2018-12-28

**Authors:** Koki Toyota, Sayo Shirai

**Affiliations:** 1 Graduate School of Bio-Applications and Systems Engineering, Tokyo University of Agriculture and Technology Naka, Koganei, Tokyo, 184–8588 Japan

The world’s population is increasing at an unprecedented rate and exceeded 7.5 billion in 2018. To meet the projected demands from not only the rising population, but also diet shifts, global crop production needs to double by 2050 (24). Several options have been proposed to solve this world crisis including the expansion of croplands, yield improvements through variety selection and better management practices, and the more efficient use of currently arable lands.

Diseases caused by fungi, bacteria, and viruses threaten crop production and cause *ca.* 10 to 20% yield losses in major crops worldwide in spite of the adoption of protection (22). Thus, proper disease control contributes to increased crop production. Many options are now available for this purpose, such as integrated control (6) and the development of new control (35) and diagnostic methods (20). Research on suppressive soils is also useful for solving yield losses caused by diseases because no or few diseases occur in certain types of soils. Disease suppressive soils have been defined by Baker and Cook (2) as “soils in which the pathogen does not establish or persist, establishes but causes little or no damage, or establishes and causes disease for a while but thereafter the disease is less important, although the pathogen may persist in the soils”. In contrast, diseases readily occur in conducive soils in which abiotic and biotic conditions are favorable to pathogens. Thus, a clearer understanding of the disease inhibitory mechanisms of suppressive soils will lead to the development of powerful tools to control diseases.

Previous studies have been conducted to elucidate the underlying disease inhibitory mechanisms and microbiological impact of suppressive soils (Fig. 1). Pathogens that have attracted attention are *Fusarium oxysporum* and *Guaeumanomyce graminis* var. *tritici* as well as plant parasitic nematodes, such as *Heterodera*, *Globodera*, *Meloidogyne*, *Rhizoctonia solani*, *Thielaviopsis basicola*, and *Verticillium dahliae* (7). Among them, suppressive soils against *G. graminis* var. *tritici*, *F. oxysporum*, and *R. solani* are well-known and have been intensively investigated.

Take-all, caused by *G. graminis* var. *tritici*, is the most economically important root disease of wheat worldwide. Take-all decline in incidence and severity of the disease is spontaneously seen after severe outbreaks of take-all during continuous wheat or barley monocultures. The research group of David M. Weller revealed that suppressive mechanisms are mediated by the accumulation of populations of 2,4-diacetylphloroglucinol (DAPG)-producing fluorescent *Pseudomonas* spp. (27). Until the 2010s, the majority of studies on disease suppressive soils were restricted to individual, specific, and beneficial microbial components, and the functions of entire resident soil microbial communities were overlooked due to the lack of comprehensive methods (14).

Mendes *et al.* (18) applied a microbiomic approach to suppressive soil that was induced by the outbreak of disease caused by *R. solani* and revealed that certain members of *Pseudomonadaceae* that produce a lipopeptide encoded by non-ribosomal peptide (NRPS) genes were the key populations in suppressive soil, while no significant variability in dominant bacterial groups was detected between disease suppressive and conducive soils. Soil microbiome investigations have since been strongly promoted (Fig. 1).

In *F. oxysporum* suppressive soils, Alabouvette (1) summarized that non-pathogenic *Fusarium* spp. were most likely involved in the suppressive properties observed. A recent microbiome study (29) revealed that fungal diversity differs between suppressive and conducive soils, and that several genera of fungi and bacteria, which are known for their activities against *F. oxysporum*, are exclusively or more abundantly present in suppressive soil.

Microbiome studies have provided important insights for related research fields on soilborne pathogens. Wilt disease caused by *Ralstonia solanacearum* has been extensively examined (34). Disease severity was low in certain types of soils (26) and in soils amended with certain types of composts (11), simple organic compounds (23), or with a biocontrol agent (21). Lee *et al.* (12) reported that *Proteobacteria*, *Acidobacteria*, *Chloroflexi*, *Verrucomicrobia*, and several *Archaea* were more abundant in soils without the disease symptoms of bacterial wilt, while another eight phyla were more abundant in soils with disease symptoms. Furthermore, a comparison of prokaryotic and eukaryotic communities revealed that several prokaryotes and eukaryotes were more abundant in soil without disease symptoms (13). These microbiome studies have successfully identified specific microbial phyla and orders, or sometimes specific genera, that are most likely involved in disease suppressive mechanisms. For example, the following genera have been identified as keystone components in disease suppressive soils: *Cheatomium* (19), *Kaistobacter* (14), *Lysobacter* (32), *Mortierella* (33), and *Pseudomonas* and *Streptomyces* (35).

Organic farming is becoming popular worldwide and has, in some cases, been proposed to contribute to the higher disease resistance of crops than conventional farming. Based on the findings of a microbiomic analysis, Takahashi *et al.* (30) demonstrated that the physicochemical properties of soils were not directly associated with the disease suppressive properties of rice seedlings against pathogenic *Burkholderia* spp.; however, bacterial populations showed greater diversities in organic soils than in conventional soils. In contrast, a comparative study on organic and intensive farming by Bonamomi *et al.* (5) indicated that the overall compositional diversity of a soil microbial community was not linked to the suppressive properties of the soil, and that some prokaryotic and eukaryotic genera positively correlated with the suppression of disease caused by *R. solani* in lettuce. A previous study conducted comparisons between organic and conventional cultivations (4), and the findings obtained revealed that the growth of four crops was greater in organic cultivations due to the suppression of diseases, whereas soil bacterial diversity was lower in organic cultivations than in conventional cultivations. Furthermore, this study indicated that the relative abundance of the metazoan phylotype in the eukaryotic population increased from 0.1% in conventional cultivation soil to 20% in organic cultivation soil, suggesting the important function of the whole soil ecosystem (4).

Reductive soil disinfestation (RSD) was firstly developed in Japan (28) and Netherlands (3) and has become popular due to the effective inhibition of various soil-borne plant pathogens and the lower load to environment (35). Microbial communities, particularly anaerobic populations, play an essential role in this disinfestation, and microbiome studies have revealed several keystone microbial components (18, 34).

Microbiome studies were popularized by their focus on human health, particularly the intestinal microbiota (9). They have since been applied not only to soils, but also to plant shoot and root microbiomes (31) and have provided important insights into microbial community functions in the biogeochemical cycles of carbon (10), nitrogen (25), and sulfur (15). A metatranscriptomic analysis may reveal functional microbial populations and genetic components in soil microbiomes. A metatranscriptomic study on the wheat rhizosphere in a disease suppressive soil for *R. solani* demonstrated that suppressive soil microbiomes more strongly expressed a polyketide cyclase and more cold shock proteins than non-suppressive soil microbiomes, while the non-suppressive microbiomes expressed many different oxidative stress genes, such as superoxide dismutase and peroxidase, which were likely induced by pathogen infections (8). Using the metatranscriptomic approach, Masuda *et al.* (16, 17) successfully detected previously unidentified keystone members involved in the biogeochemical cycles of paddy soils, such as reductive nitrogen transformation (16) and methane metabolism (17).

More than 100 years of research on suppressive soils has resulted in the accumulation of very fundamental data. Suppressive properties are mostly derived from the biological functions of soils, which will be further clarified by comprehensive microbiome investigations, excellent examples of which are introduced herein. The elucidation of microbial functions in suppressive soils will unequivocally lead to future sustainable crop production.

## Figures and Tables

**Fig. 1:**
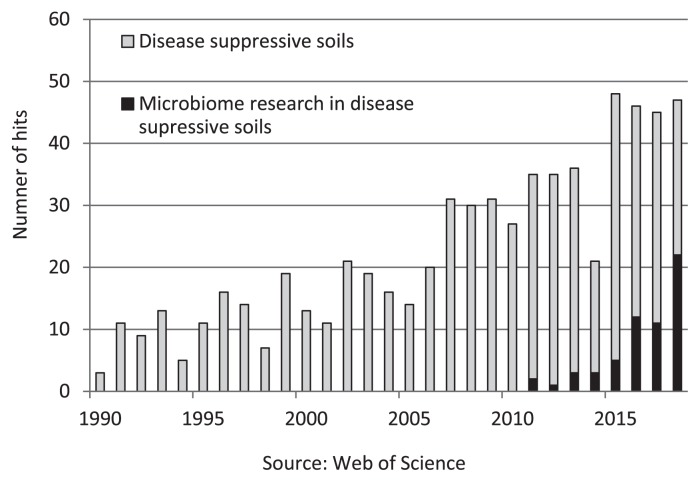
Microbiome research on disease suppressive soils. The numbers of hits (articles) are the results of key word (gray bar: disease, soil, suppressive, black bar; disease, microbiome, suppressive) searches in the Web of Science.
